# Arc-Length Characterization of Finite, Radial Growth Patterns

**DOI:** 10.1021/acs.langmuir.6c02052

**Published:** 2026-08-02

**Authors:** Andreas Andersen Hennig, Ilaria Beechey-Newman, Natalya Kizilova, Erika Eiser

**Affiliations:** † PoreLab, Department of Physics, 2152 Norwegian University of Science and Technology, Høgskoleringen, Trondheim N-7491, Norway; ‡ 49566 Warsaw University of Technology, Institute of Aeronautics and Applied Mechanics, Nowowiejska, 24, Warsaw 00-665, Poland; § V.N. Karazin Kharkiv National University, Department of Applied Mathematics, Svobody sq., 4, Kharkiv 61-022, Ukraine

## Abstract

We present a method to characterize the distribution of length scales of finite, disordered patterns with, on average, radial symmetry. This method makes it possible to quantify the distribution of characteristic length scales in cases where the conventional “linear” chord method does not work. We show that the method can clearly distinguish regular patterns, patterns that are formed by diffusion-limited aggregation, and patterns that form during the slow drying of confined, colloid-laden droplets. We also introduce a method to find the center point of these finite patterns without assuming full connectivity in the pattern. The method should be widely applicable to other finite, quasi-two-dimensional patterns, like dendritic structures, viscous fingering, liquid crystal patterns, and bacterial growth.

## Graphical abstract

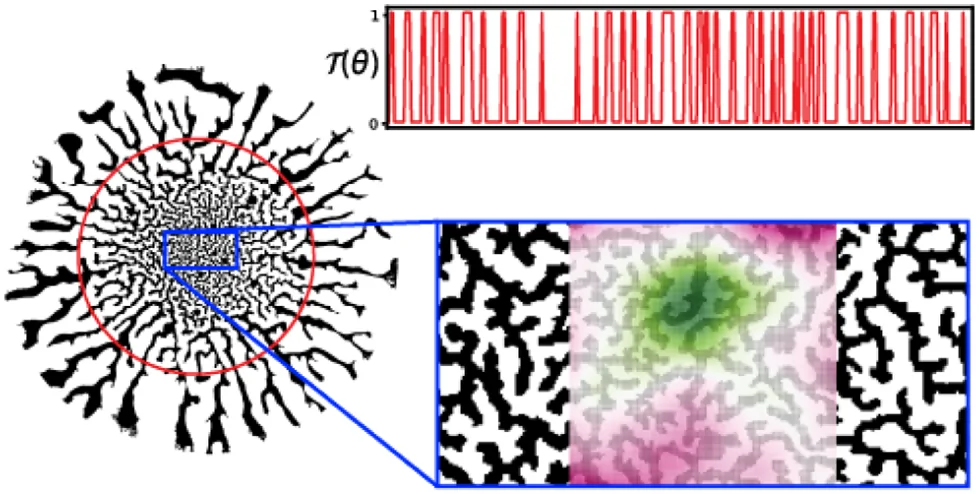

## Introduction

The so-called “chord-length distribution method”^1^ is a convenient tool to characterize the distribution of length scales of clusters and voids in a disordered yet homogeneous two-dimensional pattern of two phases (e.g., solid and air). This chord analysis aims to determine the distributions of segment lengths of any straight line that intersects a large number of solid and void spaces, e.g., in a gel network.^2^ The conventional chord analysis does not provide meaningful results for patterns that are not homogeneous and finite. Yet such patterns are important: many quasi-two-dimensional aggregation phenomena yield patterns that are (i) finite and (ii) not homogeneous in space. One example among many is the pattern formed by diffusion-limited aggregation (DLA). For such, on-average radial patterns, chord analysis does not provide a meaningful description. Here, we propose a closely related approach to probe the arc-length distribution of circles that intersect both solid and void domains. Importantly, this arc-length distribution depends on the radius of the intersecting circle, even when averaged over many similar patterns. In what follows, we will refer to this type of analysis as circular chord-length analysis; this name is less precise than arc-length distribution but stresses the analogy with the conventional chord-length distribution.

Arc-length distributions can serve as a “descriptor” of disordered radial patterns obtained experimentally, as shown in Figure 1. We use these descriptors to distinguish different *classes* of patterns. The distribution of descriptors is used in the same fashion as in a traditional chord analysis, i.e., to distinguish physical growth conditions. We compare the circular arc-length distribution for three visually different patterns (3 typical colloidal drying patterns, a diffusion limited aggregation (DLA) pattern, and a “random birthday pie” pattern) to illustrate that the method can be used to quantify the visual differences between these patterns.

**Figure 1:**
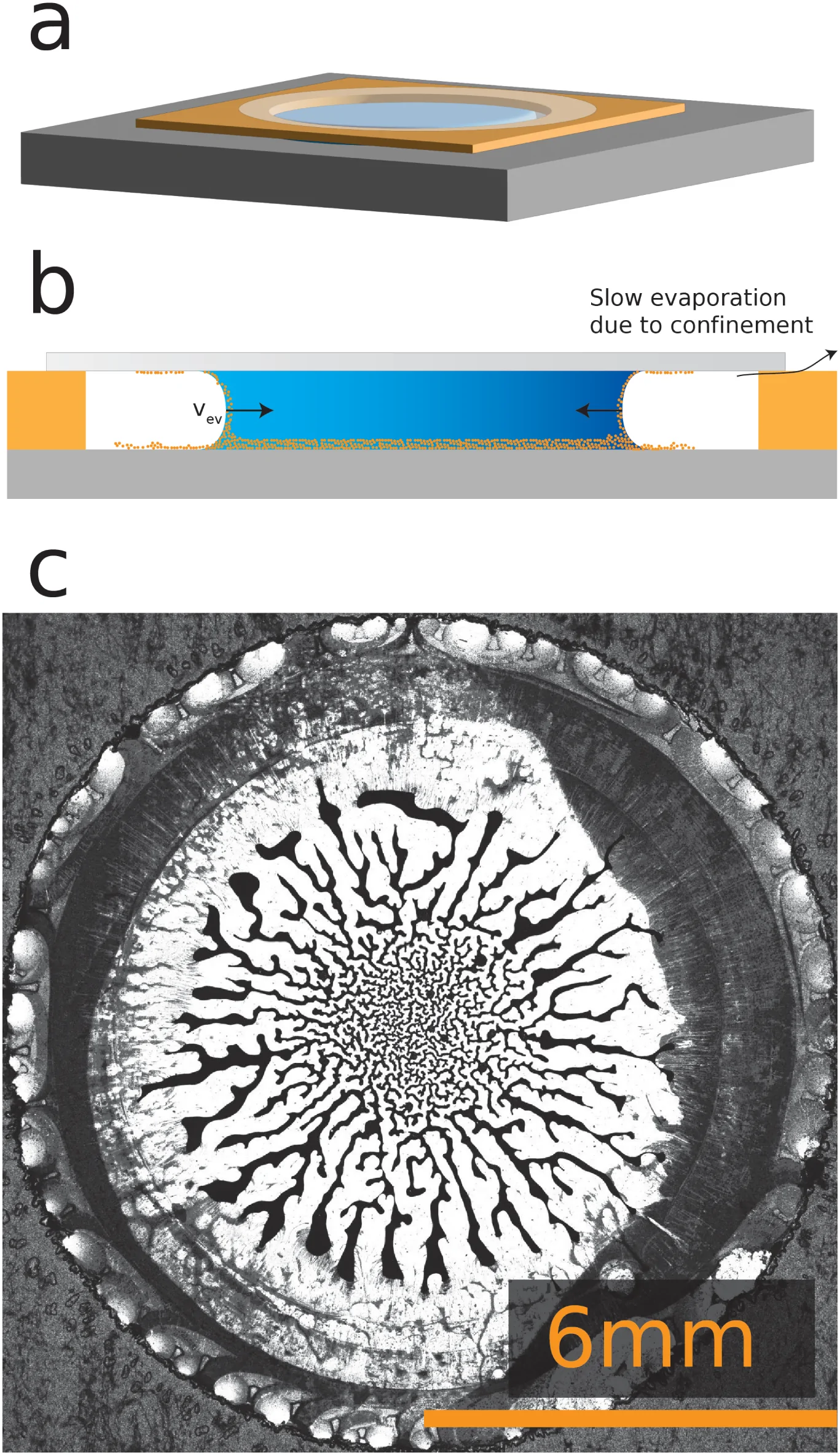
Illustration of the experimental setup from ref 8 that was used to obtain the colloidal maze-like patterns. A microscope slide is prepared with double-sided sticky tape, where we punch a hole of *r* = 6 mm and deposit a droplet of water containing 1 wt % of 1 μm-large TPM particles, as shown in (a). Then, a coverslip is placed on top of the circular cell, forcing the evaporation process to take many days. A cross-section of the cell is shown in (b), illustrating the colloid deposition on both the bottom and top glass plates. The slow evaporation process drives colloids to assemble into a contiguous maze, as seen in the photograph shown in (c).

## Systems

Roughly radial patterns often form due to the deposition of colloids, either due to evaporation of the solvent or due to (diffusion-limited or reaction-limited) aggregation of colloids from a colloid-laden suspension. A classic example is the famous “coffee-stain effect”^3^ where the dispersed coffee grounds mostly accumulate in the rim of the dried stain. The effect was explained in ref 3. for the case of droplets on partially wetting surfaces, where the droplet circumference gets pinned. As the droplet evaporates, there is a capillary flow of the solvent toward the rim, transporting almost all colloidal particles toward the perimeter. In inkjet printing, but also in coatings and paintings, this effect is unwanted, and efforts have been made to overcome the capillary flows that drive deposition on the pinned contact line.^4–7^

More recently, maze-like patterns of slowly evaporating cylindrical droplets were described by Beechey-Newman et al.^8^ These patterns form inversely ramified structures, meaning that the branches in the pattern become thinner toward the center. In the present paper, we aim to determine and compare “descriptors” of the various patterns listed above with those of simpler radial patterns.

The fractal dimension of a pattern is a convenient quantity to characterize naturally formed radial patterns that are scale-invariant. This fractal dimension can be used to distinguish different growth mechanisms; e.g., viscous fingering, stable displacement, or capillary fingers in the invasion percolation of two immiscible fluids in porous media.^9^ In contrast, the patterns observed in ref 8 are *not* scale-invariant: the characteristic length scale of the branches changes rather abruptly with radius, something that does not lend itself to a fractal or multifractal description. It is for this reason that, for such systems, we explore the angular equivalent of the chord method.

The remainder of this paper is organized as follows: first, we introduce the circular arc-length distribution method (CAL) and its relation to the linear chord-length distribution introduced by Méring and Tchoubar.^1^ We then consider examples of applications of the CAL method to experimentally observed drying patterns of colloidal droplets, to DLA patterns, and to artificial radial patterns. We show that, for experimental patterns, the CAL method can generate unique descriptors that depend on the radius of the intersecting circle. Such an analysis is particularly useful in cases where the nature of the pattern formation changes with the radius of the deposit.

## Arc-Length Distributions

The circular arc-length analysis used in this work is a fairly straightforward generalization of the linear chord-length method of Levitz and Tchoubar.^10^ In its simplest form, the CAL method is used to characterize biphasic patterns, where the two “phases” might be real phases of matter, e.g., solid and vapor, or simply different labels (e.g., black and white, characterizing an artificially generated pattern), as seen in Figure 2.

**Figure 2:**
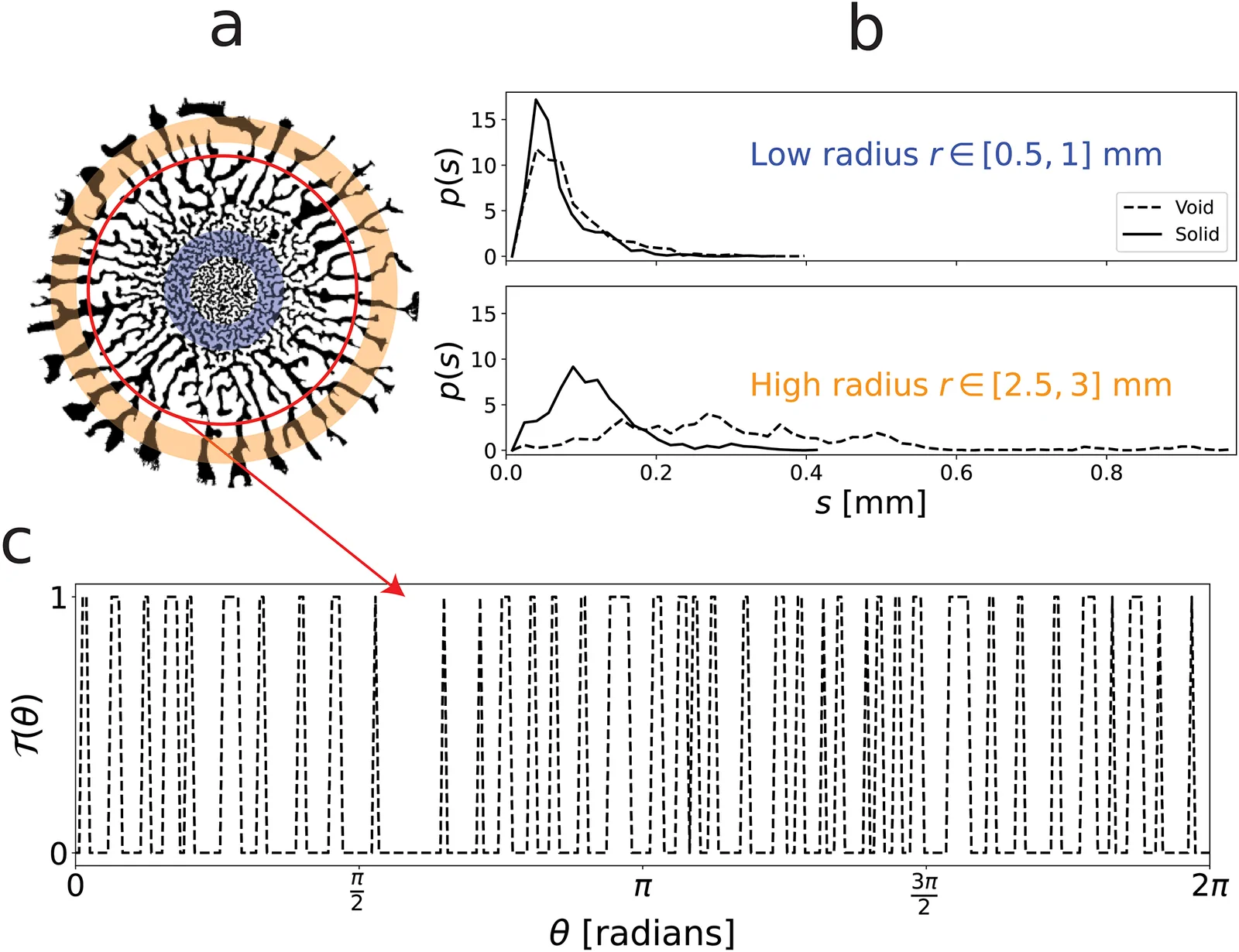
(a) Illustration of the circular arc-length distribution for the colloidal drying pattern P2, which was appropriately cropped and binarized. For a given radius (red circle), we find the angle of each white-black interface, allowing us to store the arc length *s* between each interface. (b) Resulting distribution of the arc lengths *s* between interfaces, where *p*(*s*) is the probability density for a given *s*. Radii lying in the orange shell show a broader distribution than those lying in the blue inner shell, with finer structures. (c) Telegraph signal T(θ) obtained from the particular radius illustrated in (a), with θ starting from the 3 o’ clock position and moving clockwise. In the signal, 1 indicates the solid phase, and 0 corresponds to the void phase. The details of the CAL distribution differ from sample to sample, but the overall characteristics remain the same.

We use a standard method to binarize the experimental images, similar to the method used in Zupkauskas et al.^2^ First, we apply a Gaussian blur to the image and then threshold it using Otsu’s method.^11^ Finally, a labeling algorithm is used to filter out all clusters below a certain size to minimize the random labeling errors that inevitably occur.

The first step in CAL analysis is to determine the center of the pattern. For natural patterns such as DLA, the choice of the center is obvious: it is the point at which the pattern originated. Conversely, in the case of drying patterns, the center might be identified with the last point to dry; ideally, circles around the central point should correspond to the location of the drying front at different points in time. If the drying proceeds symmetrically, then the last point to dry would be the correct choice for the central point in the CAL analysis, but in general, there is some arbitrariness in choosing the center of the pattern.

Once the center of the pattern has been located or chosen, we can draw circles of increasing radius *r* around this center. Typically, such a circle will intersect many boundaries between the two phases (say: “solid” and “void”). If we attribute the value 1 to all points in the solid phase, and 0 to points in the void phase, then we can construct a function T(θ,r) , where 0 < θ ≤ 2 π is the angle specifying a point on the circle corresponding to radius *r*. As T(θ,r) can have only the two values 0 and 1, the resulting function at constant *r* looks like a (random) telegraph signal that depends on *θ*, with an example shown in Figure 2c. In experiments, the resolution with which the pattern is obtained is limited by the pixel size, typically a square with length and width Δ. A pixel “belongs” to a given circle if that circle intersects the pixel area. In general, a pixel might belong to more than one circle. However, if the radii of adjacent circles differ by more than $\Delta \sqrt{2}$ then every pixel belongs to at most one circle. Of course, the density of pixels on a circle will not be rigorously constant, but in practice that is not a serious problem for determining arc-segment lengths.

In some cases, pixels may have the wrong “label” due to noise. Unless the noise level is very high, it is unlikely that two or more adjacent pixels will be incorrectly labeled due to noise. To remove incorrectly labeled pixels, one should check if their label differs from that of all their neighbors, i.e., if the pixel is isolated. Such pixels should be removed from the analysis. Of course, this procedure can be refined if necessary. However, a labeling error at a domain boundary cannot be removed in this way. In the cases that we study, the noise levels are so low that such errors should have a negligible effect.

Having thus identified the “random telegraph” signal on a given circle, we can identify arc segments on the circle where the label remains the same. The arc length of a segment is defined as the distance in the angle θ between two adjacent label changes. Note that, as the pattern in θ is periodic with a period 2π, the distance between the two boundaries of an arc segment should be defined as the distance (in θ) between a given segment boundary and the *nearest periodic image* of the adjacent boundary.

## Analysis of Experimental and Synthetic Patterns

We tested the CAL method by applying it to the quasi-2D colloidal drying patterns P1, P2, and P3 in Figure 3. Although these patterns were formed under similar conditions, they can differ qualitatively, as explained by Beechey-Newman et al.^8^ In addition, we applied the same CAL analysis to a numerically generated two-dimensional diffusion-limited aggregation model^12^ and an artificial pattern of random segments, which we refer to as the “random birthday pie” (RBP). The DLA pattern is generated by first initializing a particle at the center, then generating particles that diffuse freely until they diffuse into the center, where they stick. The RBP pattern is generated by first setting the total number of fingers (e.g., 10 fingers). Then, for each finger (alternating between black and white), we draw its thickness as a random number from a distribution, e.g., a Gaussian distribution with a mean of 2 and variance of 4 N(2,4) . Negative numbers are discarded. Finally, we rescale all drawn numbers such that they sum to 2π, which is the circular sector, or finger thickness, of each finger.

**Figure 3:**
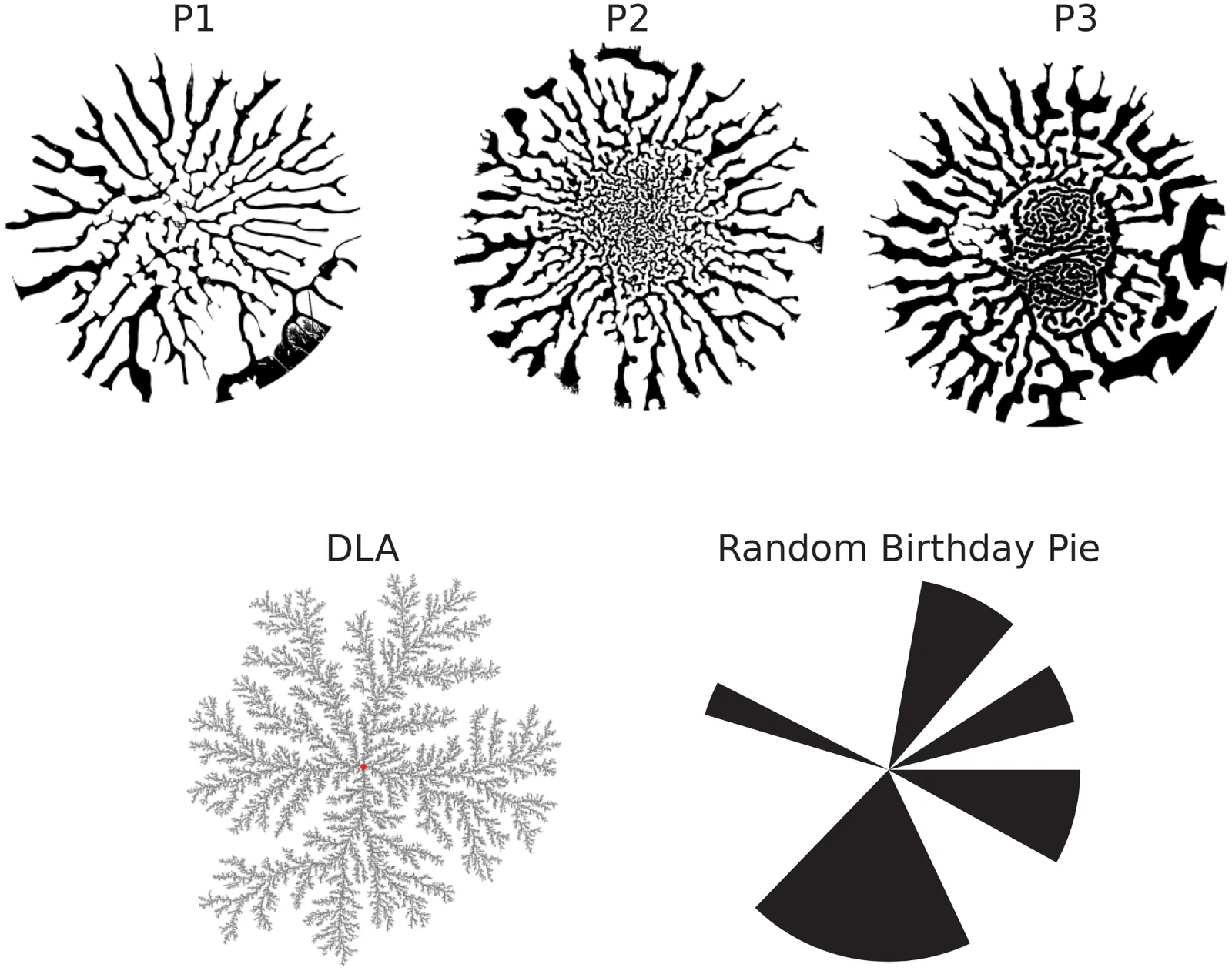
5 patterns are compared in this paper, where 3 are drying patterns obtained by the method described in Figure 1.^8^ The images were then appropriately cropped and binarized. In all 3 colloidal drying patterns, we clearly see the finger thicknesses varying, in particular in the void phase. The lower two images are of a DLA pattern, which was generated with 400,000 particles in a 2048 × 2048 grid, and an RBP pattern, with 10 segments drawn from a normal distribution N(2,4) , producing a radially invariant CAL distribution.

We used these particular synthetic patterns because they share characteristics with real experimental finger growths. Diffusion-limited aggregation patterns have a striking similarity to viscous fingers through porous media at the limit of zero surface tension.^13,14^ We also point to a different finger formation in an evaporating colloidal droplet, leading to a pattern resembling scallop shells,^15^ which resembles our random birthday pie pattern.

As discussed by Levitz and Tchoubar,^10^ DLA patterns yield a power-law distribution of the arc lengths in the void phase: *p*_p_(*s*) ∼ 1/*s* ^*d*_m_–1^ where *s* is the arc length and *d*_*m*_ is the fractal dimension of the pattern in the void phase. Below, we first apply the circular arc-length analysis to a radial colloidal drying pattern^8^ and then compare it to that of a DLA pattern. As the mechanisms by which the drying and DLA patterns form are very different, we might expect that this should result in rather different CAL distributions.

The circular arc-length distributions of the drying pattern P2 are presented in Figure 2. From this figure, we see that the distributions vary with the radius of the pattern; the goal of the method is to understand these variations. It is instructive to compare our patterns with two characteristic behaviors. In DLA, the finger thicknesses are 1 pixel, with only a few exceptions. Conversely, a radially invariant pattern, such as the RBP, has a finger thickness proportional to the radius, with a constant determined by the fraction of solid phase in the pattern: *s* = φ · *r*.

In the conventional chord-length analysis, where we create histograms of chord lengths in *x*- and *y*-directions in a porous medium, for instance, we are usually interested in finding an average characteristic length scale. This could be, for instance, the average pore size in the system.^2^ In our radial analogy, we illustrate the mean arc lengths for the experimentally obtained and artificial patterns in Figure 4. The RBP pattern scales linearly in both phases (with the solid fraction φ ≈ 0.5 as the slope; turquoise dashed lines). In contrast, the DLA pattern has a constant solid arc length, while the average *void* arc length grows as a power law with the radius (black dashed lines). We find that the drying patterns fall somewhere between the two synthetic patterns. All colloidal drying patterns have, on average, thicker fingers (solid phase) and thicker voids (void phase) at the outer radii. The main difference is that the finger thickness and void thickness are comparable close to the center, but the average void finger thickness grow much faster. These are properties that clearly distinguish the colloidal patterns from other comparable maze-like patterns, e.g., those arising from a frictional instability, characterized by a *constant* finger thickness throughout the whole pattern^16^ or Turing patterns, characterized by a single fastest growing mode.^17^

**Figure 4:**
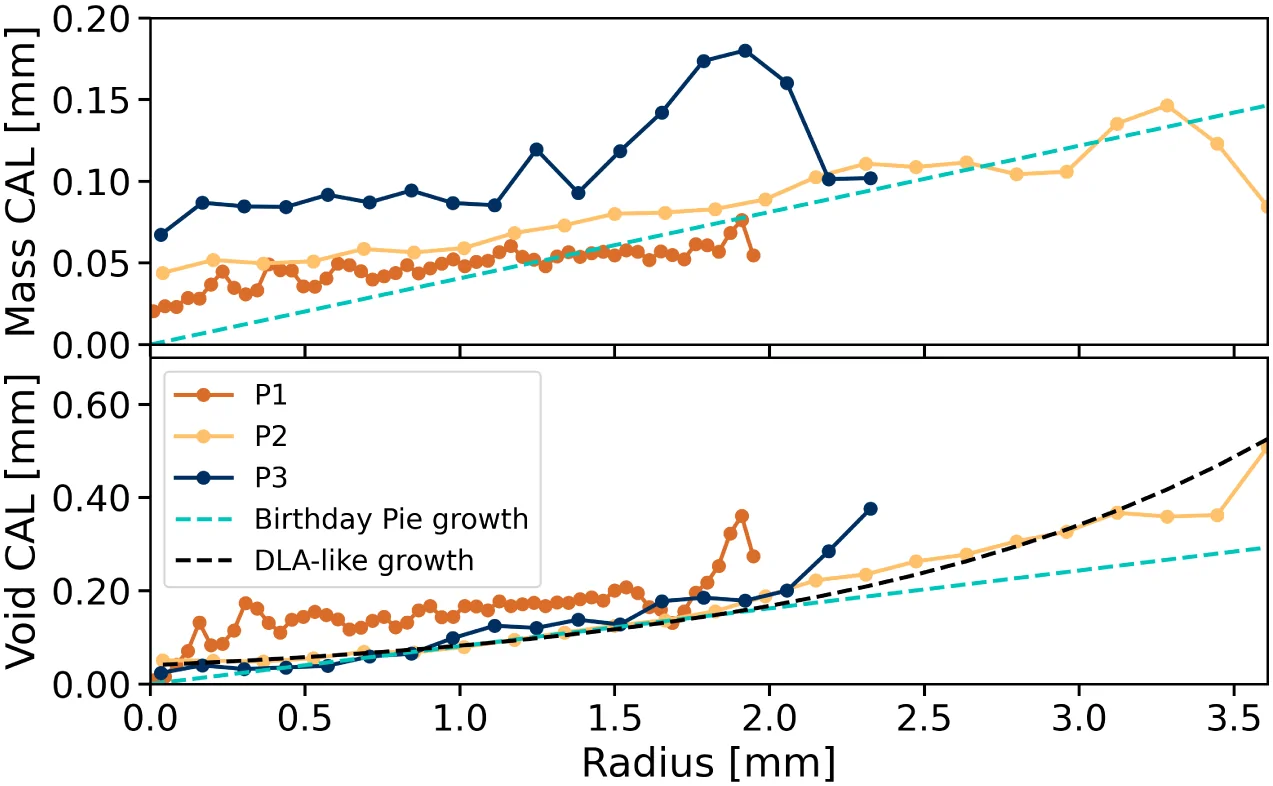
Average arc lengths of both phases, averaged over d*r* = 20 pixels, for the 3 drying patterns P1–P3. The growth trends of the cavity CAL of the synthetic pattern are added as dashed curves. However, we do not show the mass CAL for the DLA case, as this CAL is necessarily equal to one.

The exact reason why the colloidal finger thickness shrinks as the droplet evaporates is not well studied. We theorize that the change in wavelength is caused by a pressure competition between the curvature of the tip of the fingers and the osmotic pressure of the colloids. As the interface evaporates, colloids are swept up to form a crust, and at a critical crust thickness the stresses overcome the Laplace pressure, causing a secondary fingering instability. This is explained in more detail in ref 8.

We can also explore other properties of the telegraph signal we find in our radial chord-length analysis. Starting with the smallest binning length of d*r* = 1 pixel, we study three properties with respect to the radius: area fractions, number of interfaces, and cross-radius correlation functions. In Figure 5, we present the fraction of the solid phase (i.e., by counting the frequency of 1 in the telegraph signal) of the drying patterns at each radius. We expect φ = 0 far away, where we have a monolayer deposition and therefore do not have any clear fingers;^8^ likewise, we expect that the center of the pattern should be in the solid phase, and expect φ = 1 as *r* → 0. All 3 colloidal maze patterns studied display 3 different characteristics in φ­(*r*): P1 oscillates around a stable value, where the peaks in the oscillations correspond to the radii at which branching occurs; in pattern P2, the solid fraction increases as the pattern dries; pattern P3 has two fairly sharp jumps with a stable value between them. As these patterns are formed in similar conditions, the fluid instability offers a surprising sensitivity to the subtle differences in the initial conditions.

**Figure 5:**
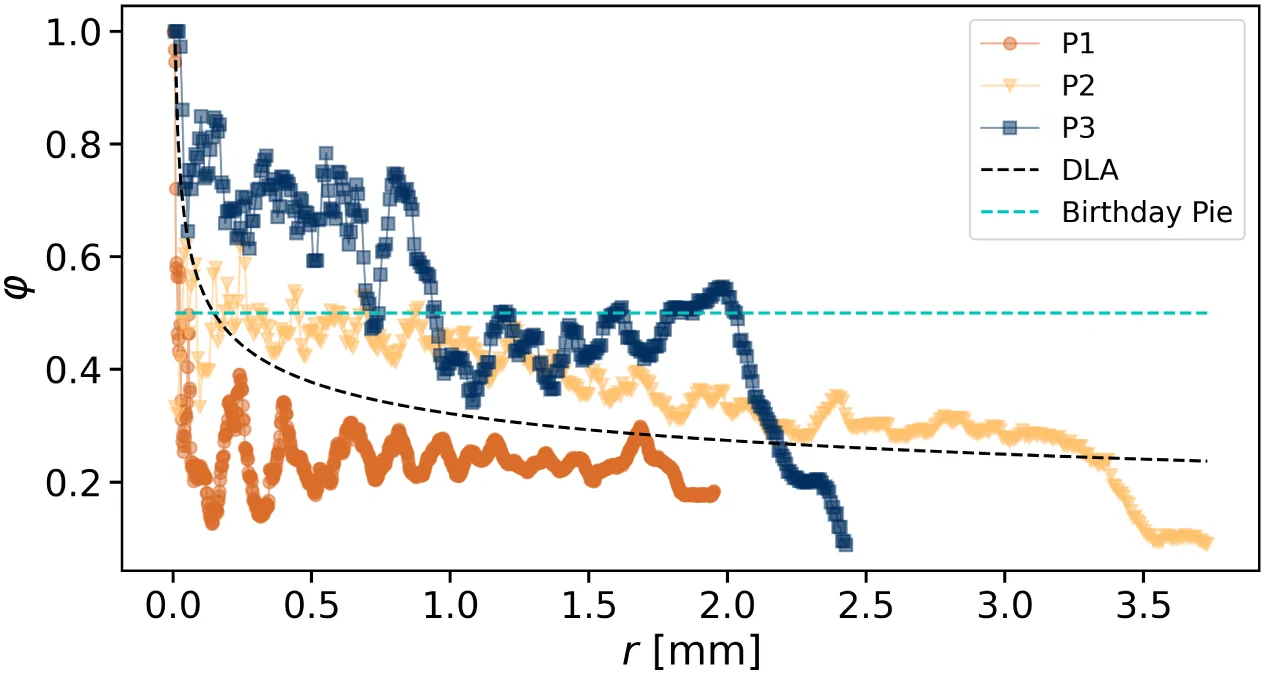
Solid fraction φ along the circular shell of d*x* = 1 at radius *r*. One means the shell contains only solid, e.g., at the center of the patterns, while 0 means the shell contains only the void phase.

At each radius, it is possible to count the number of discontinuous jumps in the telegraph signal. Each of these jumps represents a change in the phase (i.e., an interface); the lengths between phase changes are the arc lengths we study. Thus, counting the number of jumps in the signal is the same as counting the number of distinct arc lengths, and is therefore proportional to the number of fingers at each radius. The scaling is known for the DLA and Birthday Pie: DLA creates interfaces as a power law with its fractal dimension *N* ∼ *r* ^(*D*_f_–1)^,^18^ while a radially invariant pattern has the same number of interfaces at every radius. The phase jumps of our drying patterns are shown in Figure 6 and display a transition between the characteristic behavior of DLA, close to the center, and RBP far away from the center. The fingers clearly merge as the droplet dries, while the fluid instability causing the fingers also forms new fingers during drying. The scaling in region (II) in Figure 6 means the rate of merging roughly matches the rate of forming new fingers. At some point, there is a transition where the fingers merge more frequently, leading to region (I), where the colloidal fingers follow a power-law ″DLA-like″ decay toward the center. We tried to fit the phase shifts to a simpler logarithmic fit but did not find good agreement with the experimental data.

**Figure 6:**
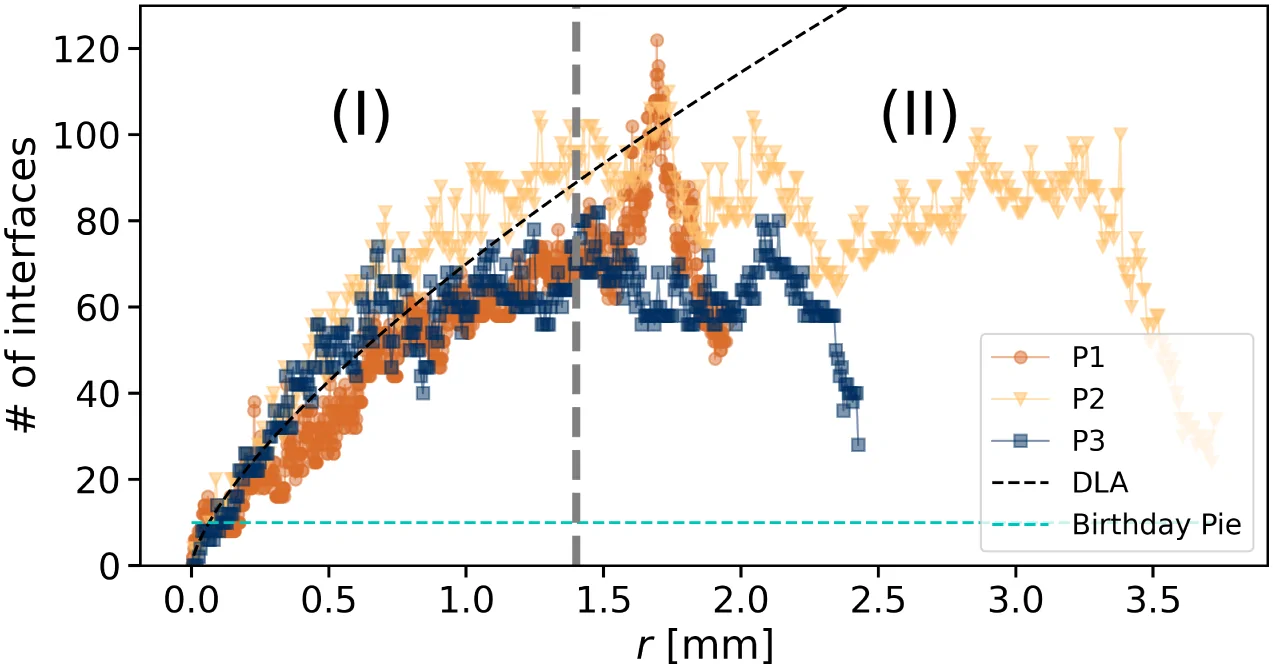
Number of phase shifts of the telegraph signal, corresponding to the surface area the fingers generate. In a DLA, the number of interfaces grows with the circumference, while radially invariant patterns, e.g., random birthday pies, have a constant number of phase shifts. We mark this figure with two regions where the colloidal patterns exhibit different scaling: Region (II) is characterized with a fairly stable number of interfaces; at some point, the fingers start merging faster than they are formed, and in Region (I) there is a fairly stable merging rate across the drying patterns.

We may also study the correlation between telegraph signals to quantify the structural similarity between radii. Here, we want to factor out the correlation between solid fractions φ, so we can compare only the position of the fingers. We therefore remove the average from the telegraph signal and only look at the fluctuating part $\mathrm{T̃}\left(r,\theta \right)=T\left(r,\theta \right)-\langle T\left(r,\theta \right)\rangle_{\theta }$ , with $\langle A(\theta \rangle_{\theta }=\frac{1}{2\pi }\int_{0}^{2\pi }A\left(\theta \right)d\theta$ . The cross-correlation function of the pattern is defined as

1 $$C\left(r,r^{\prime }\right)=\frac{{\langle \mathrm{T̃}(r,\theta )\mathrm{T̃}(r^{\prime },\theta )\rangle }_{\theta }}{\sqrt{{\langle \mathrm{T̃}{\left(r,\theta \right)}^{2}\rangle }_{\theta }{\langle \mathrm{T̃}{\left(r^{\prime },\theta \right)}^{2}\rangle }_{\theta }}}$$

A pattern that is radially invariant, such as the RBP patterns, will have the property that $T\left(r,\theta \right)=T\left(r^{\prime },\theta \right)$ ; these are perfectly correlated at all radii, with only a small numerical error due to rasterization of the image. We can see that the drying patterns P1–P3 decorrelate faster close to the center and are more persistent in the outer regions, where the fingers initially form. We then use eq 1 to find the average correlation of the separation of the compared signals as *C*(Δ*r*) = ⟨*C*(*r*,*r*′)⟩_|_ *_r_*_–*r*′|=Δ*r*_. We plot these in Figure 7, where the decorrelation of the 3 drying patterns collapses to the same exponential decay curve. We introduce the persistence length ξ as the length where the signal has decorrelated by a factor 1/*e*, *i.e. C*(Δ*r*)=exp­(-*r*/ξ).

**Figure 7:**
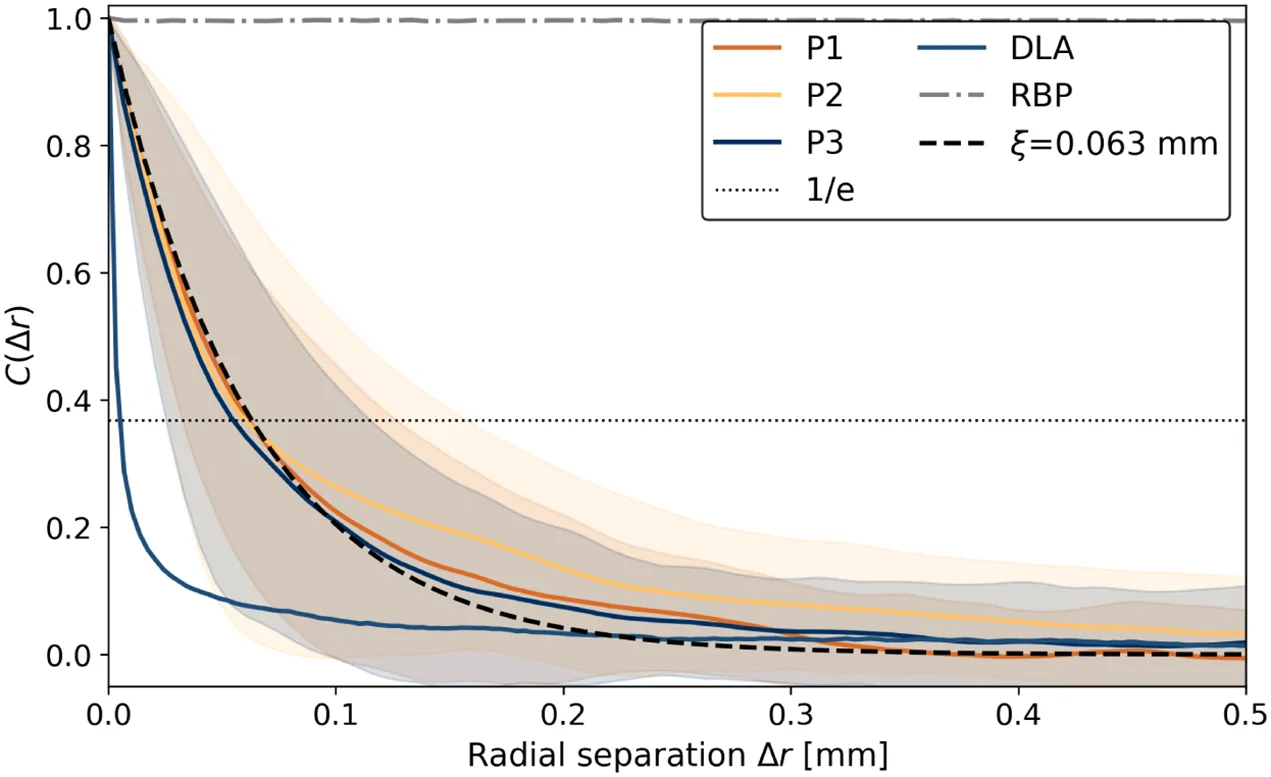
Average correlations of telegraph signals, by separation distances Δ*r*, for all 5 patterns. The RBP remains perfectly correlated throughout the pattern, while the DLA decorrelates rapidly—with a correlation length of 2 pixels. The drying patterns P1–P3 decay exponentially, with decay lengths of 0.063, 0.065, and 0.061 mm. The average decay length is plotted as ξ = 0.063 mm. The correlation functions for P1, P2, and P3 shown in this figure are quite noisy (the noise band is colored homogeneously). The curves for *C*(Δ*r*) correspond to the average.

The persistence length is found to be 0.063 ± 0.002 mm for the drying patterns, which translates to about 35 colloid diameters. Physically, this is how long the fingers tend to stay aligned and is therefore a characteristic length between the branching points. The two chosen synthetic patterns are clearly different in persistence lengths from the evaporated colloidal patterns. Since the telegraph signal of the Random Birthday Pie is independent of radius, its persistence length is infinite. On the contrary, the DLA pattern is formed by particles randomly diffusing to the cluster, forming a fractal. Thus, on average, the position of a particle in a DLA finger is only dependent on the position of the last particle in the finger. Thus, we find a decorrelation length of 2 pixels in our DLA clusters.

Likewise, we may also find the correlation between angles in the telegraph signal, where the arc length is the natural variable to compare the decorrelation. We find that these decorrelation lengths give similar results as the mean arc lengths, as shown in Figure 4.

## Estimating the Colloidal Concentration from Arc Lengths

When inspecting the fingers under a microscope, it is clear that they are fairly uniform in colloid density across the pattern, while the space between the fingers contains no colloids at all. The fingers consist of colloids on both the bottom and the top substrates, with no colloids in between. The bottom layer is packed with slightly more than a monolayer (1+*ε* layers), while the packing in the top substrate has a few holes, roughly equaling the excess (i.e., 1-*ε* layers).^8^ Therefore, we may ask the question: Can we relate the simple 2D pattern, easily captured with a camera, to the colloidal mass deposited?

We start by approximating the fingering pattern as exactly 2 monolayers of colloids consisting of randomly packed monodisperse spheres with a density of ρ_TPM_ = 1.314 g cm^–3^.^19^ We assume a packing fraction of a monolayer of random close-packed disks ϕ_2*D*_ = 0.772 ^20^ leading to an effective 3D packing fraction ϕ = ϕ_2*D*_ · *V*_sphere_/*V*_cylinder_ = 0.513. The thickness of the monolayer is the colloid diameter *d*_c_ = 1.8 μm. Now it is possible to relate the information on solid fractions φ­(*r*) in Figure 5 to the deposited colloidal mass at radius *r*

2 $$m_{c}\left(r\right)=\rho_{\mathrm{TPM}}\cdot 2\phi \cdot d_{c}\int_{0}^{r}\varphi \left(r^{\prime }\right)\cdot 2\pi r^{\prime }dr^{\prime }$$

This relation between the mass and radius of a pattern is useful for probing the fractality of the patterns. A fractal will have a power-law relation between the mass deposition, scaling with its fractal dimension *m*_d_(*r*) ∼ *r* ^*D*_f_^.^9^ In comparison, the scaling of the RBP is simply its Euclidean dimension *m*_*d, RBP*_ = φπ*r* ^2^. We find that the mass scaling of patterns P1–P3 is well described by power-law fits, meaning the patterns may be described as fractals, with a fractal dimension similar to that of the DLA pattern (see Figure 8). We also measure the fractal dimension with a box-counting method: we cover the image with squares of size δ and count the number of squares that contain any solid-phase pixels—denoted *N*—and we repeat this for δ from the smallest possible size (the pixel size) to the largest (comparable to the total pattern radius), spanning roughly 2 decades of box sizes. The number *N*(δ) follows a power-law relation, with the box-counted fractal dimension as the exponent *D*_f_ = ln*N*/lnδ. A more detailed explanation is found in ref 9. The results of the two methods applied to the colloidal patterns are summarized in Table 1. These values are fairly similar to the fractal dimension of a DLA pattern,^12^ except for the mass fractal dimension of P1, which is closer to that of an RBP. We may now relate the deposited mass to the colloids deposited in the fingers. As the droplet shrinks, it can be split into its fingers and its “inscribed circle” of radius *r* as in ref 8. The mass of the colloids in this evaporating droplet must be the same mass we find in the fingers between the center and *r*, and can be calculated with eq 2. We compute the weight of the water as *m*_w_(*r*)=ρ_w_π*r* ^2^ *h*_*cell*_, with the value *h*_cell_ = 80 μm. Finally, we compute the colloidal concentration in the evaporating droplet as it shrinks $C\left(r\right)=\frac{m_{c}\left(r\right)}{m_{w}\left(r\right)+m_{c}\left(r\right)}$ , which is compared to the initial colloid concentration *C*_0_ and shown in Figure 8. Interestingly, we note that the instability leading to finger formation occurs at different initial (average) colloid concentrations: at *C*(*r*_max_)/*C*_0_ = 0.70 for P1, 0.88 for P2, and 1.29 for pattern P3. We may also estimate the initial mass of the colloids. The initial concentration is 1 wt %, and the cell dimensions are *r* = 6 mm and *h*_cell_ = 80 μm. Thus, the total mass of the colloids *m*_c, tot_ is roughly 100 μg. The evaporating colloidal systems are found to initially deposit colloids in a uniform monolayer (seen as gray color inside the cell in Figure 1c), until there is a transition to the finger instability.^8^ We can assume the droplet *either* leaves colloids in the initially forming monolayer, *or* in the fingering pattern. Thus, by taking *r*’ = *r*_max_ in eq 2, we find the mass of colloids going into the fingering pattern. We compare this weight to the initial colloid weight and find the percentage of colloids going into the fingers to be 7% for pattern P1, 31% for P2, and 19% for pattern P3.

**Figure 8:**
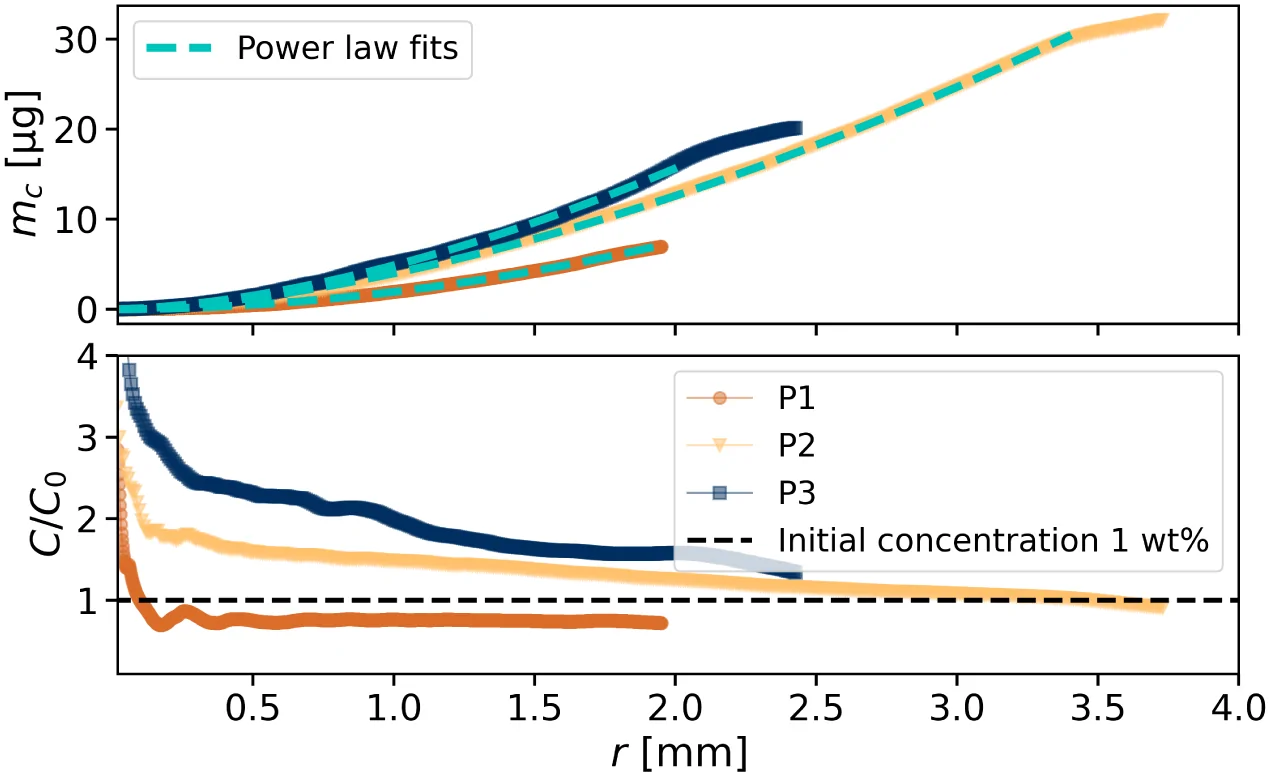
A plot of the cumulative colloid mass *m*_c_(*r*) deposited in the pattern-forming stage of the evaporation process. This is found by assuming a uniform colloidal packing fraction throughout the pattern, left in exactly 2 monolayers. We find that the mass deposition scales with a power law, with exponents found in Table 1. We then relate the deposited mass to the colloid concentration of the droplet as it is evaporating *C*(*r*), compared to the initial concentration *C*_0_ = 1 wt % for the patterns P1–P3. The initial concentration of the droplet is illustrated with a dashed black line.

**Table 1:** Comparison of the Fractal Dimensions of the Patterns P1–P3, Measured with Two Different Methods^a^

	Mass scaling *D*_f_	Box-counting *D*_f_
P1	1.95	1.61
P2	1.67	1.68
P3	1.69	1.72

a The mass scaling method is based on a power-law fit to the mass deposition data, while the box-counting method measures how many boxes *N* of size δ we need to cover the full pattern, with the fractal dimension found as *D*_f_ = ln*N*/ln*δ*.

## Using the Telegraph Signal to Find the Center

The choice of the location of the center of a roughly radially symmetric pattern is, to some extent, arbitrary. The simplest method to find the center of mass of a pattern is to locate the center of mass. However, for the colloidal drying patterns, the center of mass is skewed by the transverse thick fingers forming at the rim of the pattern. These thick fingers tend to form unevenly; hence, the center of mass is clearly different from any visually selected center. If the pattern is a fully connected network, like a DLA, it is possible to follow the fingers toward their center. Following the idea of Horton and Strahler on complexity in river networks,^21,22^ we may skeletonize a pattern and count the number of bifurcations at each node in the skeleton network. However, this method may provide results that are clearly off the center, as it (a) requires a fully contiguous pattern and (b) causes arbitrariness where there are multiple equally ordered points.

In order to resolve the arbitrariness of deciding the center, we propose a method using the arc-length distribution. First, we define a test center *X*_t_, where we may use any of the mentioned approaches as a first approximation. We define a window of *N*  × *N* pixels centered around *X*_t_. We then decide the range of relevant radii, *r* ∈ [*r*_min_, *r*_max_]. For each pixel, *X*_*i*_ within the window, we sum the telegraph signal as

$$T_{S}\left(\theta ;X_{i}\right)=\frac{1}{r_{\max}-r_{\min}}\int_{r_{\min}}^{r_{\max}}T\left(r,\theta ;X_{i}\right)dr$$

We then calculate the power spectrum of the signal as $G\left(\mathrm{\theta ̂}\right)=\int_{0}^{\infty }|T_{S}\left(\theta ;X_{i}\right){|}^{2}d\theta$ where *θ̂* denotes the Fourier mode of the angle. A clear periodicity in the summed telegraph signal will result in a sharp peak in the power spectrum. The minimum entropy center is generally the point that generates the clearest angular periodicity in the summed telegraph signal. We therefore use the power spectrum to define an information entropy as $S\left(X_{i}\right)=-\int_{0}^{\infty }G\left(\mathrm{\theta ̂};X_{i}\right)\ln G\left(\mathrm{\theta ̂};X_{i}\right)d\mathrm{\theta ̂}$ . The best center, based on our information entropy, is simply picked as the extremal point $X_{c}={\mathrm{argmin}}_{X_{i}}\left(S\left(X_{i}\right)\right)$ . Figure 9a-c shows a comparison of two potential centers for the RBP figure, along with their power spectra. Summing the spectra as an entropy, we obtain the map in Figure 9d. In Figure 9e the comparison between the minimum entropy, center of mass, and Horton-Strahler approaches shows that our minimum entropy determination of the figure’s center is the most accurate.

**Figure 9:**
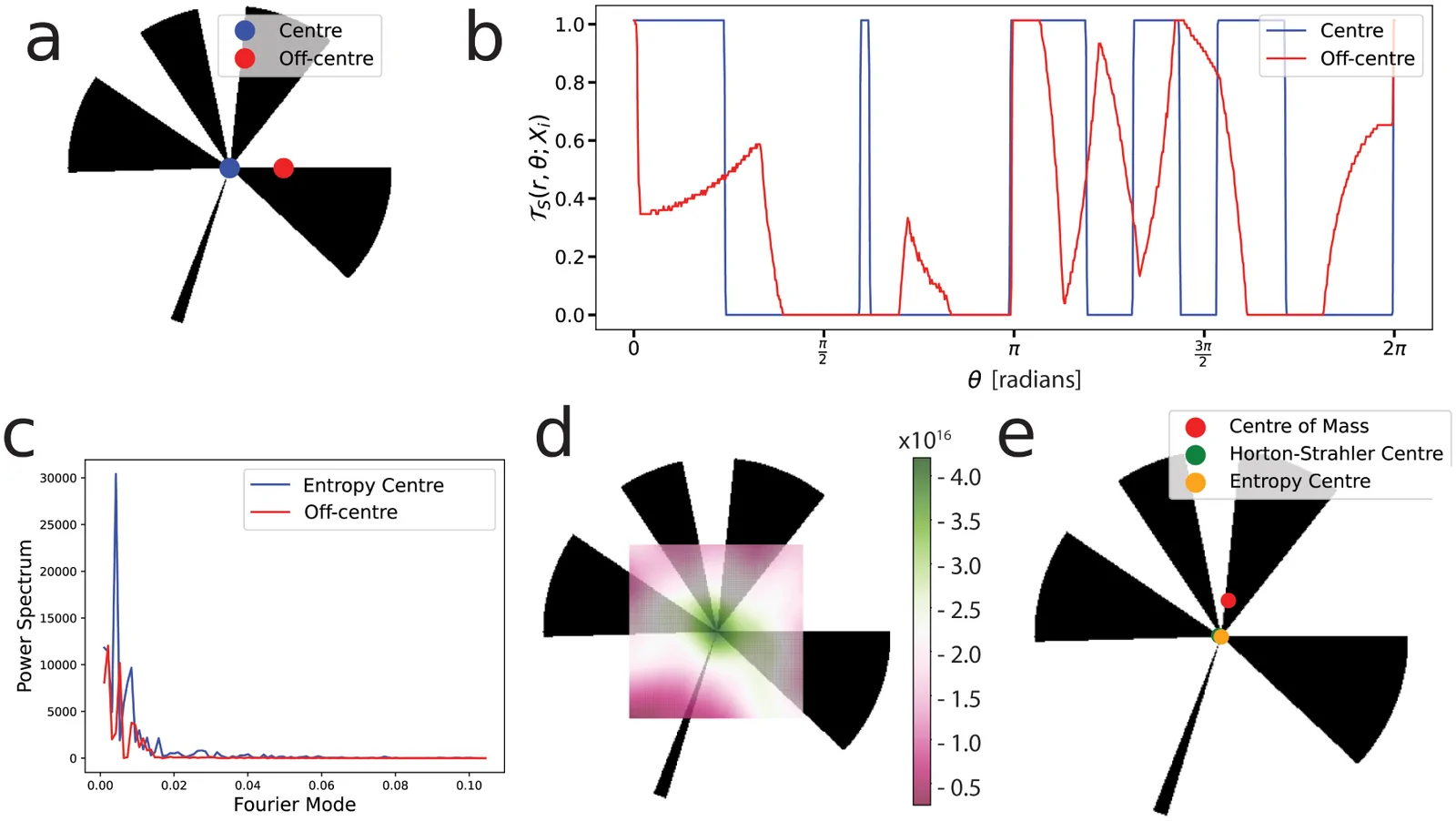
(a)–(c): A comparison of two points, where one is in the center of the Random Birthday Pie pattern and the other is placed 50 pixels to the right, as shown in (a). (b) compares the summed and normalized telegraph signal $T_{S}\left(\theta ;X_{i}\right)$ from the two points, and (c) shows their respective power spectra, which are used to calculate the entropy. (d) Shows a map of the calculated entropies at each point in the selected window, and (e) shows the center calculated with 3 different methods.

The RBP and DLA patterns have well-defined centers, so we can quantify the errors; these are presented in Table 2.

**Table 2:** Comparison of the Errors We Find in the Center of Mass and Entropy Methods^a^

	Center of Mass	Entropy
DLA	9.53 px	22.34 px
RBP	16.92 px	0.84 px

a For the DLA we use 20 generated patterns of 100,000 particles and a max radius of 1024 pixels. For the random birthday pie we generate 20 random patterns with 10 fingers and a max radius of 150 pixels.

To find the best center to apply our CAL analysis of samples P1 and P2 is not straightforward. Depending on experimental parameters, such as possible pinning centers on the support surfaces or how the samples are leveled horizontally, the apparent center may not be located at the center of the sample chamber. However, we can choose a center by eye and then obtain the maps of the local entropies. These are shown, along with a comparison with other methods, in Figure 10. Clearly, the green region is the most representative of the true center of the labyrinthian pattern of our samples. Hence, our minimum entropy approach helps to find the best center, around which the resulting CAL analysis delivers the most reliable results for comparisons with other growth laws.

**Figure 10:**
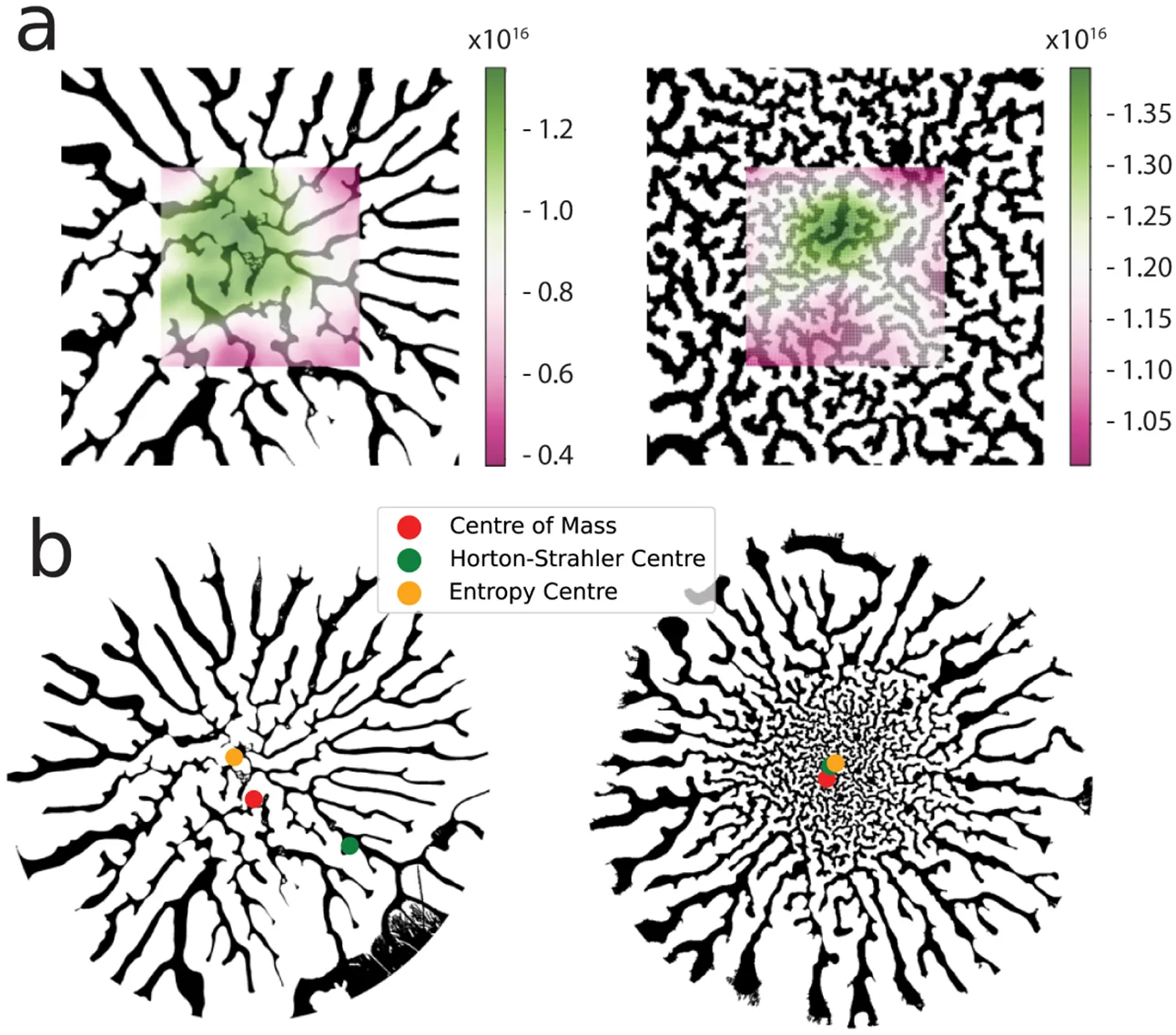
(a) Resulting entropy map of patterns P1 and P2, using the method presented in this paper. Green in the scale bar corresponds to the best location of the center. (b) Resulting center points, compared with the two alternative methods.

## Conclusions

We find that extending the traditional chord-length distributions to roughly radially symmetric patterns enables us to characterize these patterns in a way that is not feasible with the traditional method. The distributions may serve as descriptors of the patterns, which vary from experiment to experiment due to subtle differences in the exact initial conditions.

We find that the colloidal drying patterns P1–P3 fall somewhere between the characteristic DLA and Random Birthday Pie patterns. Most importantly, we find that the competition between the generation and merging of fingers sustains a fairly stable number of fingers, up to a point where the fingers merge more rapidly. The finger thickness, however, clearly changes with the radius from the center, distinguishing these colloidal patterns from the DLA and other well-known models. We compute the correlation between the telegraph signals and relate them to a characteristic exponential decay with a persistence length of ξ = 0.063 mm, or roughly 35 colloid diameters. With a few assumptions, we are also able to relate the information from the arc lengths to describe how the pattern deposits its colloidal mass and to the colloid concentration of the droplet as it evaporates. This is also used to characterize the fractality of the patterns, showing that the fractal dimension is similar to that of DLA. Finally, we use the arc lengths to map an information entropy on the pattern, which we use to select a center in cases where the position of the center is not obvious from the preparation protocol.

Hence, the method can be applied to patterns that are not simply radially invariant or self-similar but also to intermediate cases. A natural extension of this work is to use our method to study the effect of specific experimental parameters, such as the colloid concentration, fluid viscosity, or cell height. In addition, we think this work could be extended to study vastly different circularly symmetric finite patterns, e.g., viscous fingering through porous materials at various capillary numbers, frictional fingers of sedimented particles,^16^ and patterns emerging from photophobic microalgae.^23^

## Data Availability

All the images, methods, and Python codes used in this paper are available in a public Zenodo repository.^24^ This includes the method used to generate the synthetic patterns (Diffusion-Limited Aggregation and Random Birthday Pies), the method we used to binarize our experimental images, and the full, annotated Python codes we developed to perform our analyses.
